# Numerical Analysis and Experimental Study on Fabrication of High Aspect Ratio Tapered Ultrafine Holes by Over-Growth Electroforming Process

**DOI:** 10.3390/mi10120824

**Published:** 2019-11-27

**Authors:** Yunyan Zhang, Pingmei Ming, Runqing Li, Ge Qin, Xinmin Zhang, Liang Yan, Xinchao Li, Xingshuai Zheng

**Affiliations:** Institute of Non-Traditional Machining & Equipment, Henan Polytechnic University, Jiaozuo 454000, China; yun9404@163.com (Y.Z.); 18300622369@163.com (R.L.); zhangxm@hpu.edu.cn (X.Z.); yanliang@hpu.edu.cn (L.Y.); hpulixinchao@163.com (X.L.); xshuai_zheng@163.com (X.Z.)

**Keywords:** ultrafine tapered hole, over-growth electroforming, high aspect ratio, electroforming, precision nebulizer plate

## Abstract

High aspect ratio (HAR) ultrafine tapered holes (diameter ≤5 μm; AR ≥5) are the most important elements for some high-tech perforated metallic products, but they are very difficult to manufacture. Therefore, this paper proposes a nontraditional over-growth electroforming process. The formation mechanism of the HAR ultrafine tapered holes is investigated, and the factors controlling the geometric shape evolution are analyzed numerically. It was found that the geometric shape and dimensions of the holes are highly dependent on the diameter and thickness of the photoresist film patterns, but are hardly affected by the spacing between two neighboring patterns; the achievable diameter for a given hole depth becomes small with the increasing pattern diameter, but it becomes big with the increasing pattern thickness. These correlations can be well interpreted by the established two empirical equations that characterize the relationship between the minimum orifice of the tapered hole and the structural parameters of the photoresist film patterns previously formed on the substrate. Application of the fabricated 1500 tapered holes with 3-μm diameter and 17-AR as the nozzles of the medical precision nebulizer is also examined. The studies show that the over-growth electroforming process is highly applicable in fabricating the perforated metallic plate with HAR ultrafine tapered holes.

## 1. Introduction

Tapered microholes are frequently regarded as the crucial elements for some high-tech perforated products, such as inkjet printing nozzle plates, filtration media, printing nickel screens, surface-mount technology stencils, shaver foils, nebulizer plates, etc. Thus, these holes are required to have well-defined geometric shapes, dimensional accuracies, and surface quality to favorably meet their application requirements. However, these requirements are very difficult to be simultaneously met during manufacturing, especially for the high aspect ratio (HAR) ultrafine holes (diameter is less than 5 μm; AR is larger than 5) such as medical nebulizer nozzles, which need to have well-defined tapered or conical geometric profiles. 

Up to now, several techniques have been exploited to fabricate these specially shaped holes: jet electrochemical machining [1], chemical or electrochemical etching [2,3], electro discharge machining [4], laser beam machining [5], and electroforming [6]. Although each of these techniques has its own unique advantages to shape them, comparatively, the electroforming technique is thought to be the most competitive one due to its diverse superi-orities, including ultrahigh dimensional accuracy, almost no cracks and burrs, smooth surface morphologies, accommodation to mass production, very small thermal stress, etc. Therefore, electroforming has been developed to be one of preferred techniques to create high-quality ultrafine hole products. For manufacturing these apertures, there are two electroforming modes: thick resist through-mask electroforming [7,8,9] and over-growth electroforming [10,11]. The thick resist through-mask electroforming technique produces perforated components by inversely duplicating the geometric shape of the patterned, insulating resist molds, which are finally removed. As a result, the quality of the through-mask electroformed holes including their surface morphologies and geometrical designs are highly dependent on the patterned molds. However, the duplicating mechanism of the through-mask electroforming makes it extremely challenging to create the HAR ultrafine holes with extraordinary three-dimensional shapes such as a bell shape and trumpet shape mainly because the patterned molds can hardly be correspondingly achieved. In contrast, over-growth electroforming is a process that does not completely follow the electrodeposition duplicating mechanism as the through-mask electroforming does. It forms the geometric shape of the holes majorly by over-growing electrodeposited metal over the insulating thin-film patterns on the conductive substrate. Theoretically, the surface of the over-grown electroforms is naturally produced and thus is changed smoothly. In addition, the over-growth electroforming is able to form the tapered hole with a very big size difference between its two orifices and a very high aspect ratio, which is hardly produced by the through-mask electroforming. This is because during over-growth electroforming, one orifice is continuously enlarged, and in contrast, another orifice is shrunken as the hole depth is increased. Consequently, the over-growth electroforming was selected as a promising process to create HAR ultrafine tapered hole components [12,13,14,15,16,17]. Nevertheless, the evolution mechanism of the geometric shape of the tapered holes and its affecting factors are rarely reported, and thus have not been well understood yet. Furthermore, the factors that determine the orifice sizes of the tapered hole are also little clarified. For most applications, the obtained minimum orifice size is extremely important, because it fundamentally determines the size of the achieved resulting particle or feature. 

The evolution mechanisms of the features formed by over-growth electrodeposition processes have been investigated for some other applications. Yang et al. [18] fabricated metal microelectrode arrays via over-plating technology and prepared the microelectrodes with different shapes by changing the structural parameters of the insulating film patterns. Sun et al. [19] manufactured the mold for microlens production by the over-growth electrodeposition process, and analyzed its affecting factors. In this paper, the evolution mechanism of the HAR ultrafine tapered holes used for the medical nebulizer plate during over-growth electroforming was studied, and the correlations between the orifice size of the tapered holes and the structural parameters of the photoresist film patterns were also established in the form of equations based on experimental and numerical simulation data, so that they could be well used for practical applications. 

## 2. Numerical Analysis 

### 2.1. Over-Growth Electroforming Principle

**Figure 1:**
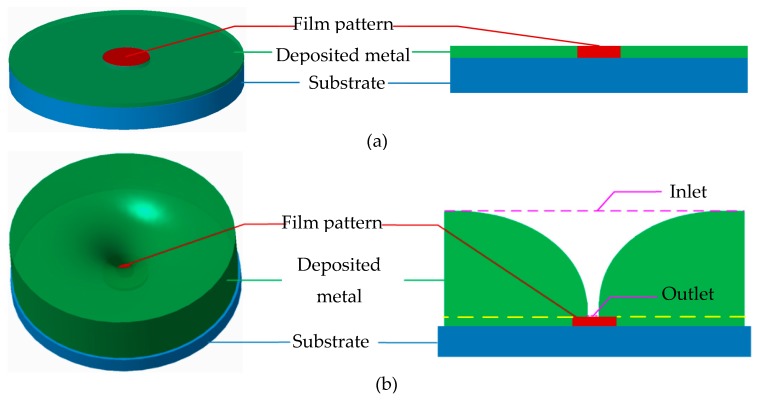
Schematic diagram of the over-growth electroforming process for forming a high aspect ratio (HAR) ultrafine tapered hole. (**a**) Normal electroforming phase; (**b**) Over-growth electroforming phase.

**Figure 2:**
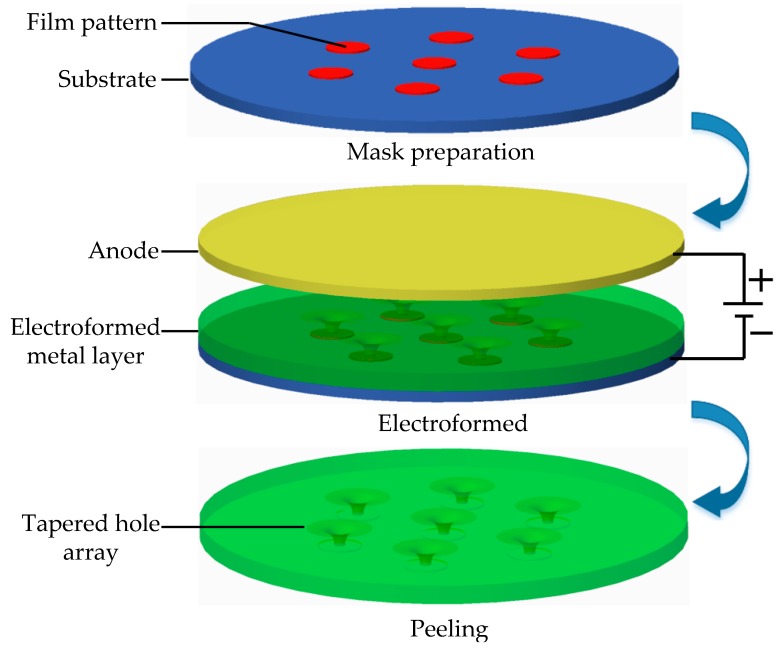
Main steps of fabricating the tapered hole plate by the over-growth electroforming process.

Figure 1 and Figure 2 show the schematic diagram of the formation of the ultrafine tapered holes by the over-growth electroforming process. Metal electrodeposition during the over-growth electroforming is carried out on the conductive substrate on which some insulating thin photoresist-film circular patterns are previously prepared. In the early stage, deposition occurs only on the blank areas (without a covered patterned film) of the substrate, and the deposit grows only in a vertical direction (i.e., the through-mask electroforming phase) until its thickness equalizes the thickness of the film patterns, following the electroforming duplication law. Then, the deposit begins to grow in all directions (i.e., the over-growth electroforming phase), which means that the deposit also stretches laterally over the patterned film while growing vertically. In this way, tapered holes with a gradually reduced orifice facing the substrate are created as the deposition process continues.

Therefore, it can be deduced from the formation procedure of the tapered hole by the over-growth electroforming process that the resulting geometric shape and dimensions of the hole could be highly dependent on the thickness and area of the film patterns, the deposit thickness, and the spacing between two neighboring film patterns. To make the tapered holes with the desired geometric shape and high tolerance dimensions, the above-described structural parameters of the film patterns need to be carefully designed. Figure 2 shows schematically the main steps of fabricating a tapered hole plate via the over-growth electroforming process. These steps include: (a) preparation of the photoresist film patterns on the conductive substrate, (b) electroforming, and (c) separation of electroformed plate from the substrate.

### 2.2. Model Development and Numerical Solution

**Figure 3:**
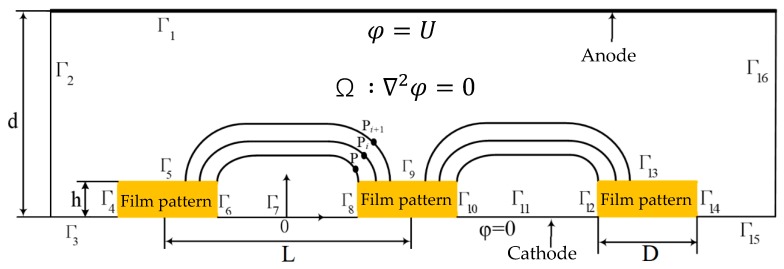
Diagram of the simulation model for electroforming a tapered hole.

To facilitate numerical simulation of the metallic deposit growth during through-mask electroforming and the over-growth electroforming process, a two-dimensional (2-D) model was developed, as shown in Figure 3. In the model, the pattern thickness, h, pattern diameter, D, spacing of two neighboring patterns, L, and interelectrode gap between the anode and the cathode, d, were defined. In order to analyze the possible influences of the aforementioned parameters on the geometric shape and dimensions of the formed hole and further to establish relevant quantitative equations, some of them were varied during simulations: h was set to be 5 μm, 3 μm, and 1 μm; d was selected to be 30 μm, 50 μm, and 70 μm; and L was set to be 75 μm, 100 μm, and 150 μm. 

To simplify the calculation and simulation operations but without loss of generality, the following assumptions were made [6]:

- the potential of each electrode surface is equal, ignoring the boundary effect;
- the electrolyte is isotropic and its conductivity is constant in the enclosed area Ω;
- regardless of electrochemical polarization and concentration polarization;
- the electric field between the electrodes approximates to a steady electrostatic field at a special moment.

According to the electric field theories, the electric potential (Ø) distribution in the domain (Ω) is governed by Laplace’s equation:

(1) $$\nabla^{2}\varphi =\frac{\partial^{2}\varphi }{\partial X^{2}}+\frac{\partial^{2}\varphi }{\partial Y^{2}}=0$$

where *X* and *Y* are the location along the x-axis and y-axis in the interelectrode gap, respectively.

The boundary conditions for electrodeposition are:

(2) $${\varphi |}_{\Gamma_{1}}=U\;\left(at\;anode\right)$$

(3) $${\varphi |}_{\Gamma_{3}}={\varphi |}_{\Gamma_{7}}={\varphi |}_{\Gamma_{11}}={\varphi |}_{\Gamma_{15}}=0\;\left(at\;cathode\right)$$

(4) $${\frac{\partial \varphi }{\partial n}|}_{\Gamma_{i}\left(i=4,5,6,8,9,10,12,13,14\right)}=0.$$

Based on the above four equations, the electric field distribution in Ω can be obtained. Moreover, the current density, J, at a specific point, which can be calculated from the field Ω, is stated as Ohm’s law,

(5) J=κE

where *κ* is the conductivity of the electrolyte solution and *E* is the electric field strength.

Then, the instantaneous electrodeposition velocity, *v*, is expressed as

(6) v=ηωJ=ηωκE

where η is the current efficiency, is the volume of the metal electrochemical equivalent (cm^3^/(A·s)).

An orthogonal coordinate system is established in which the origin of the coordinates is set to be at the midpoint of the two neighboring patterned films. Set the point *P*_0_ (*x*_0_, *y*_0_) at the cathode surface at the initial time of the electrodeposition process (*t* = 0), after a period, *i*Δ*t*, of electrodeposition, the point *P*_0_ (*x*_0_, *y*_0_) moves to the point *P_i_* (*x_i_*, *y_i_* ) and then moves to the point *P_i_*_+ 1_ (*x_i_*_+ 1_, *y_i_*_+ 1_) after a time step, Δ*t*. The coordinate relationship between *P_i_* and *P_i_*_+ 1_ can be expressed as

(7) $$\{\begin{array}{c} x_{i+1}=x_{i}+v_{ex}\Delta t=x_{i}+\eta \omega E_{x}\Delta t\\ y_{i+1}=y_{i}+v_{ey}\Delta t=y_{i}+\eta \omega E_{y}\Delta t \end{array}$$

where *E_x_* and *E_y_* represent the electric field strength along the x-axis and y-axis, respectively.

Based on Equation (7), the point *P_i_* (*x_i_*, *y_i_*) at any time can be obtained. Therefore, if all the concerned points *P_i_* (*x_i_*, *y_i_*) at a given time are calculated and then are connected together, the profile of the deposit at that time will be achieved. According to the superposition principle of electrodeposition, if the original shape of the cathode substrate is given, the profile of the electrodeposit at any moment can be achieved by simulations. The simulations were stopped when the minimum diameter of the aperture reached the desired value. To understand the mechanisms and obtain the optimal formation parameters of the aperture by electrodeposition, numerous simulations were carried out by changing some of the key parameters (patterned film thickness, patterned film diameter, spacing between two neighboring patterned films and electroforming time). In the simulation, the required electroforming thickness was obtained by adjusting the electroforming time. The main parameters needed for the simulation operations are listed in Table 1.

## 3. Results

An SUS 304 stainless steel plate (75 mm in diameter, 1 mm in thickness) was used as the substrate on which insulating photoresist film patterns (thickness: 1 μm, 3 μm, or 5 μm) were previously prepared using a standard photolithography process. The electrolyte compositions and electrodeposition process parameters are listed in Table 2. A DC power supply was employed to the electroforming process, and the applied voltage was kept at 0.5 V. The electroforming time was varied according to the requirements of the deposit thickness. 

**Figure 4:**
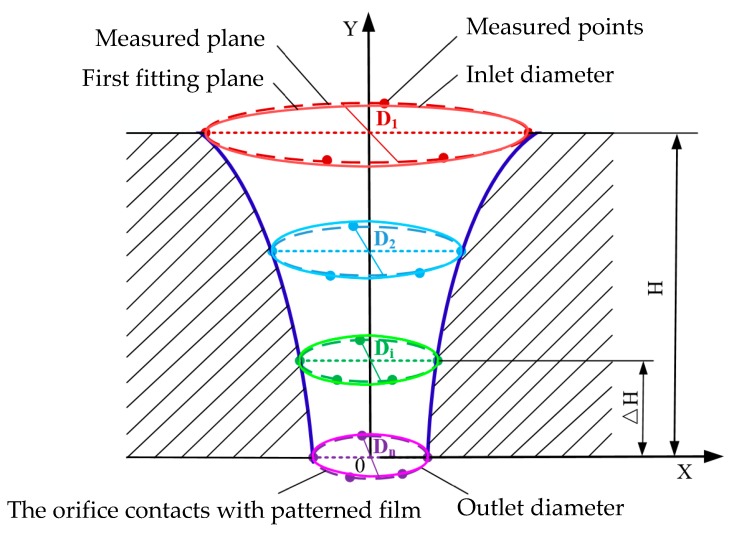
Schematic diagram of the method for measuring hole diameters and fitting the geometric profile of the electroformed tapered hole.

The geometric profile of the electroformed tapered hole was obtained by fitting the data, which were measured by the tool measuring microscope (176-591DC, MITUTOYO, Kawasaki, Japan). As shown in Figure 4, the measuring method and steps were as follows.

- Determine the whole depth, H, of the formed hole, and then divide it into n (n = 6–8) parts.
- Along the depth direction of the hole, define n + 1 measuring planes which are parallel to the substrate surface, and the first measuring plane is designated as the surface of the deposit contacting directly the substrate.
- Acquire the coordinate values of at least five different points that are located at the measuring circle formed by transecting the hole with the measuring plane, and then fit the values into a circle based on the least square method, thus calculating the diameter of each fitted circle.
- Form the three-dimensional geometric profile by connecting all the above fitted circles.

Here, we define the orifice of the hole that contacts with the substrate during electroforming as the outlet of the tapered hole, and the other orifice as the inlet.

Scanning electron microscopes (Merlin Compact, Carl Zeiss NTS GmbH, Jena, Germany; JEOLJCM6000, Tokyo, Japan) and a digital microscope (VHX-S90BE, KEYENCE, Osaka, Japan) were employed to characterize the features of the formed apertures.

## 4. Results and Discussion

### 4.1. Morphologies and Geometric Profiles

**Figure 5:**
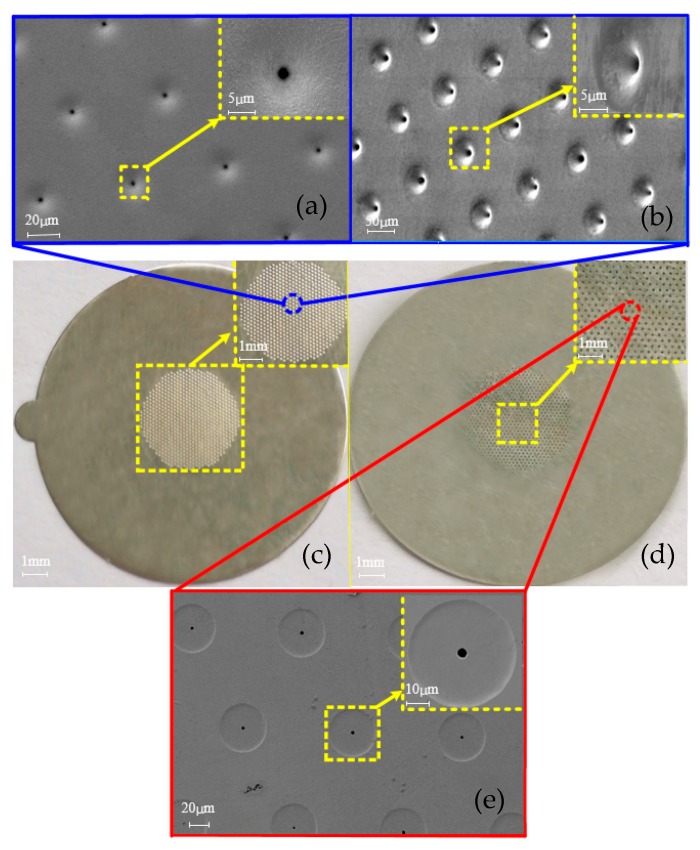
The photo and SEM pictures of the tapered hole plate formed by the over-growth electroforming process. (**a**) The SEM picture of the inlets of the holes; (**b**) the enlarged SEM pictures of the inlets of the holes observed with a 300 angle of inclination; (**c**) the photo of the inlet side of the tapered hole plate; (**d**) the photo of the outlet side of the tapered hole plate; (**e**) the SEM picture of the outlets of the holes.

The electroformed ultrafine tapered holes show a well-defined three-dimensional geometric profile whose average outlet diameter is about 3 μm and average inlet diameter is approximately 50 μm, as shown in Figure 5. These ultrafine tapered holes appear to be uniformly distributed on the plate, and their geometric profiles are of high similarity in shape with a very smooth surface. The plate sample with 1500 ultrafine tapered holes distributed within the 4 mm-diameter area shown in Figure 5 was produced based on the following parameters: the film pattern thickness h = 1 μm, the film pattern diameter D = 50 μm, the spacing between two neighboring patterns L = 100 μm, the applied voltage being constantly 3 V, and the electroforming time being 4 h.

### 4.2. Effect of Film Pattern Thickness, h, on the Geometric Shape of the Ultrafine Tapered Hole

**Figure 6:**
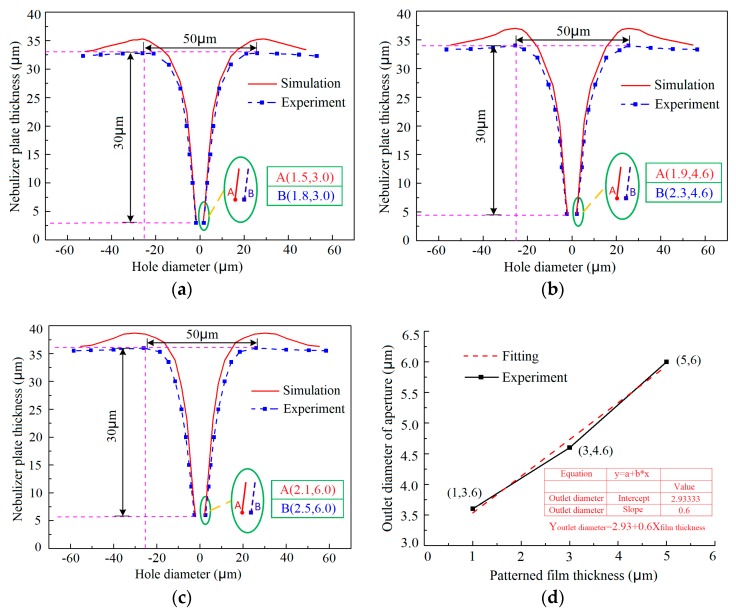
Variations of the geometric profiles and outlet diameter of the tapered hole with the film pattern thickness, h. (**a**) h = 1 μm, D = 50 μm, and L = 100 μm; (**b**) h = 3 μm, D = 50 μm, and L = 100 μm; (**c**) h = 5 μm, D = 50 μm, and L = 100 μm; (**d**) relationship between the outlet size of the hole and the film pattern thickness.

As shown in Figure 6a–c, although the film pattern thickness is increased, the three-dimensional geometric shape of the holes formed both experimentally and by simulation is highly identical, showing a sharply tapered shape. However, the measured outlet diameter of the holes, which determines the achievable minimum size of atomized particles, is gradually enlarged, from 3.6 μm to 6.0 μm, with an increasing the pattern thickness, from 1 μm to 5 μm. The simulated data of the outlet diameter showed a similar change trend. These findings revealed that the use of thinner film patterns benefits the obtainment of a smaller outlet of the tapered holes when a constant thickness nebulization plate is desired. By fitting the measured data, an empirical formula describing the relationship between the outlet size of the tapered hole, *Y_outlet diameter_*, and the film pattern thickness, *X_pattern thickness_*, is given as follows:

(8) $$Y_{outlet\;diameter}=2.93+0.6X_{pattern\;thickness}.$$

From this formula, one can determine the required film pattern thickness according to the designed outlet size of the nebulizer nozzles.

**Figure 7:**
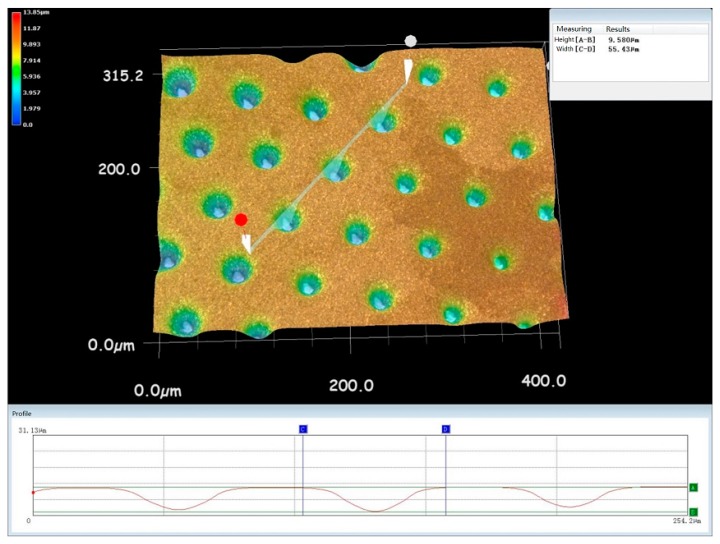
Three-dimensional (3D) topography of the tapered hole plate formed by over-growth electroforming with h = 3 μm, D = 50 μm, and L = 75 μm.

Comparing the simulation results with the experimental results, some differences can be found. The real outlet of the tapered hole is a bit larger than the simulated one. This is because in the simulation, the current efficiency in the nickel electrodeposition was assumed to be 100%, while in the real electroforming process, it is smaller than 100%. As a result, under the conditions where the electrodeposition time and the current density are both constantly given, a higher current efficiency generally means that more metal is deposited, leading to a smaller outlet. Another obvious difference between them is that in the simulated geometric shape, a humped deposit can be observed surrounding the inlet of the tapered holes, while it almost disappears in the electroformed ones regardless of the thickness of the film patterns used, as shown in Figure 7. It can be found that there is no concave region but rather a smooth transition between the adjacent two tapered holes, and the mutual influence effect between the adjacent two holes is small. This could be caused also by the assumption made for the simulations in which the current efficiency was set to be 100% and the deposit can completely duplicate the shape of the film patterns on the substrate. Therefore, theoretically, the hump is thought to be the duplicate of the step formed between the film pattern and the substrate. In effect, according to the electrodeposition theories, the electrodeposit can be gradually self-leveled with the growth of metal.

### 4.3. Effect of Film Pattern Diameter, D, on the Geometric Shape of the Ultrafine Tapered Hole

**Figure 8:**
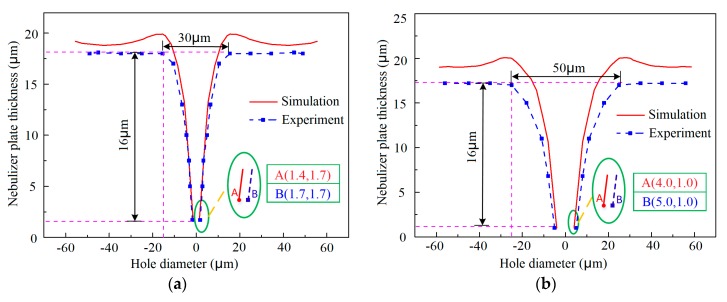

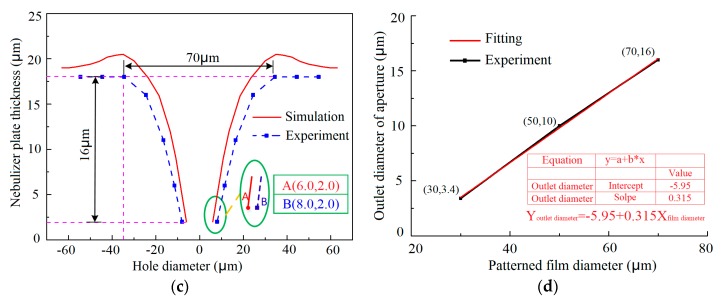
Variations of the geometric profiles and outlet diameter of the tapered apertures with the patterned film diameter, D. (**a**) D = 30 μm, h = 3 μm, and L = 100 μm; (**b**) D = 50 μm, h = 3 μm, and L = 100 μm; (**c**) D = 70 μm, h = 3 μm, and L = 100 μm; (**d**) Relationship between the outlet diameter and the patterned film diameter.

The variation of the geometric shape of the tapered hole with the film pattern diameter was shown in Figure 8a–c. The data from both simulations and experiments showed that the inlet and outlet of the tapered holes are both enlarged with the increase of the film pattern diameter when the deposited plate thickness is kept constant. The reason is that more space that is not be occupied by the over-grown metal can be left if the film pattern diameter is bigger under the same electroforming process conditions. 

As for the outlet of the tapered hole, the relationship between its size and the film pattern diameter applied can be expressed as an empirical formula by fitting the measured data

(9) $$Y_{outlet\;diameter}=-5.95+0.315X_{pattern\;diameter}$$

where *Y_outlet diameter_* is the outlet diameter of the tapered hole and *X_pattern diameter_* is the film pattern diameter. It can be seen from this formula that in order to achieve an outlet as fine as possible, a small film pattern diameter should be selected. It was also shown that the simulated geometric shape of the tapered hole is very similar to the experimental one, but in general, the electroformed hole is a bit bigger than the simulated one, which is mainly because the real electrodeposition rate is smaller than that assumed in the simulation.

### 4.4. Effect of Spacing between Two-Neighboring Film Patterns, L, on the Geometric Shape of the Tapered Hole

**Figure 9:**
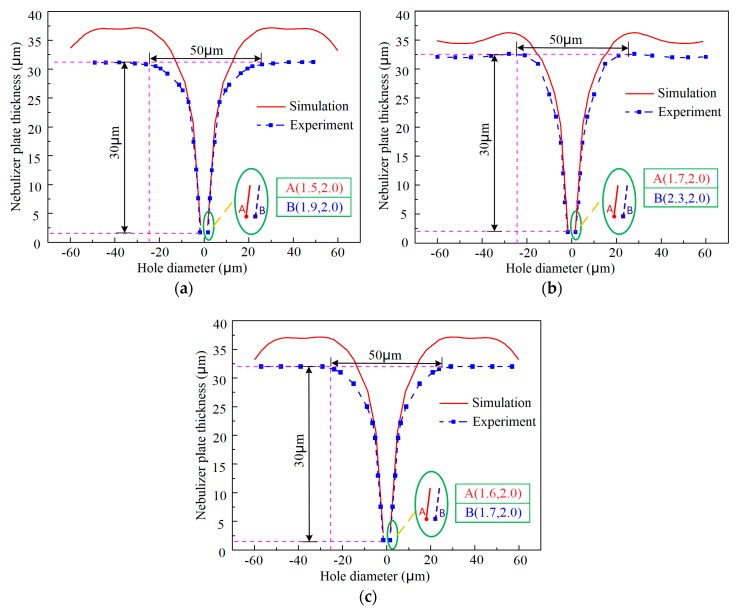
Variations of the geometric profiles and dimensions of the tapered aperture with the spacing between two neighboring films. (**a**) L = 75 μm, h = 3 μm, and D = 50 μm; (**b**) L = 100 μm, h = 3 μm, and D = 50 μm; (**c**) L = 150 μm, h = 3 μm, and D = 50 μm.

**Figure 10:**
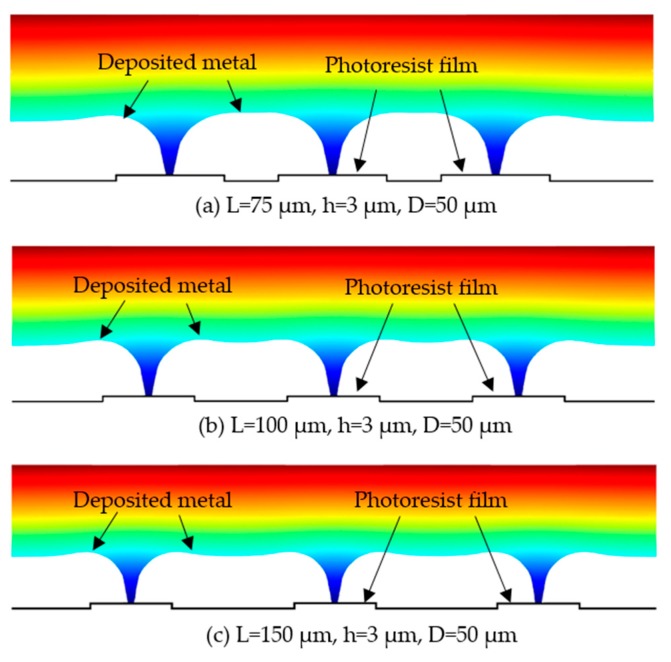
Simulation results of the tapered aperture profile with the different center distances of the ptterned films. (**a**) L = 75 μm, h = 3 μm, and D = 50 μm; (**b**) L = 100 μm, h = 3 μm, and D = 50 μm; (**c**) L = 150 μm, h = 3 μm, and D = 50 μm.

Figure 9 illustrates the variation in the geometric shape of the tapered hole with the spacing between two neighboring film patterns. From the simulated data, it was shown that the spacing has almost no effect on the outlet size of the tapered holes, which is nearly the same regardless of the change of the spacing, but it does show a great influence on the geometric profile and size of the inlet, which vary with the spacing. These changes can be further illustrated in Figure 10. Figure 10 shows the simulation results of the tapered aperture profiles with the different center distances of the patterned films. With the increasing of the distances of the patterned films, the interaction between the inlet profiles of the tapered holes was reduced. However, a concave region can be observed between the adjacent two tapered holes, and as the spacing increases, the concave region is also gradually widened because of the insulating properties of the film patterns. However, from the experimental results, it was found that the outlet of the tapered holes changes with the applied spacing without a definite change rule, while the whole geometric profile and size of the inlet hardly vary with the spacing, as shown in Figure 9. These experimental findings indicated that the desired ultrafine tapered holes can be achieved only when an appropriate spacing value is adopted. This could be because the over-growth process of the metal deposit, which determines the geometric shape of the tapered hole, is somewhat affected by the surrounding metal deposition. Therefore, if the outlet size of the tapered hole is highly concerned for practical applications, the spacing between two neighboring film patterns should be optimally chosen.

## 5. Atomization Evaluation of the Fabricated HAR Ultrafine Tapered Hole Plate

**Figure 11:**
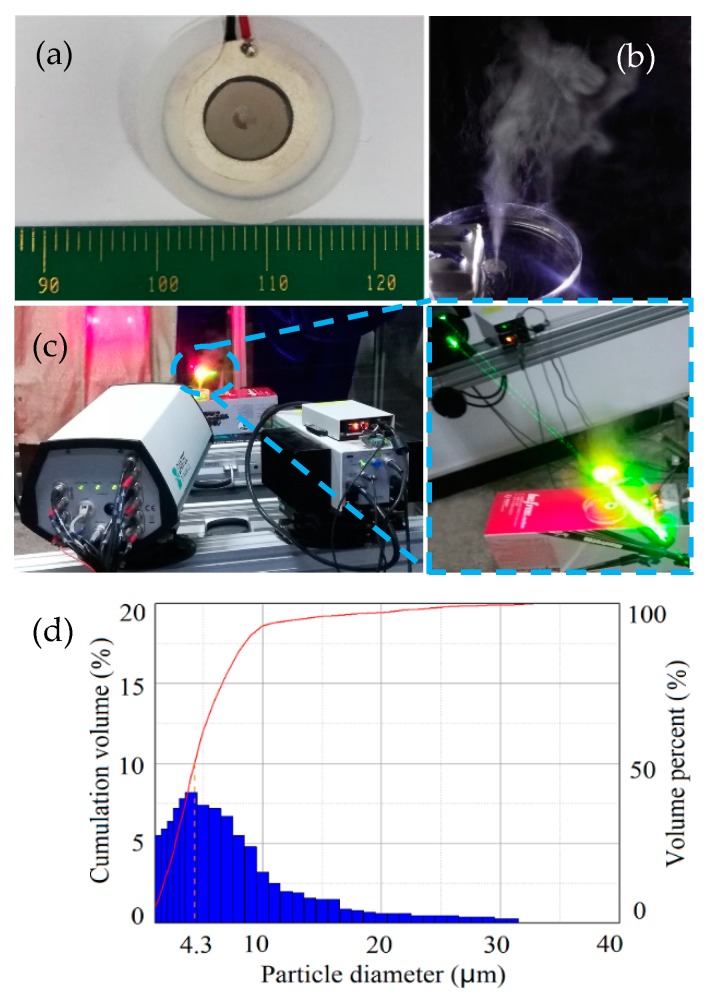
Atomization effect of nebulizer plate and the size distribution of particles using the nebulizer. (**a**) The prototype of the nebulizer; (**b**) The atomization effect of the nebulizer; (**c**) Measuring the nebulizer atomization effect by the phase Doppler particle analyzer (PDPA); (**d**) The size distribution of particles using the nebulizer.

To evaluate the atomization effect of the nebulizer plate electroformed with the optimized process conditions and parameters, atomization tests were carried out. As shown in Figure 11a, a ring-shaped piezoelectric ceramic (dimensions: Ø15 mm × Ø8 mm × 0.6 mm; material: PZT-4F) was bonded to the nebulizer plate using a conductive adhesive, and two very thin, bare copper wires were connected to the two electrodes of the piezoelectric actuator (a resonant frequency of about 108 kHz). The operation was conducted at a voltage of 5 V and a current of 0.5 A. The main parameters of the tested nebulizer plate were: the nebulizer plate is 50 μm in thickness with 1500 ultrafine tapered holes uniformly distributed within the 4-mm diameter atomization area, the hole’s average outlet diameter is about 3 μm, the average inlet diameter is approximately 50 μm (AR = 50/3 ≈ 17.3), and the pitch size between two hole centers is 100 µm. The test experiments were implemented using a phase Doppler particle analyzer (PDPA) (Dantec Dynamics Technology Corp., Copenhagen, Denmark.). The sizes of the particles generated by the nebulization process was characterized by the mass median aerodynamic diameter (MMAD). The testing result showed that the particles with a MMAD of less than 4.2 μm are able to be atomized from the tested nebulizer plate, which means that the cumulative volume (less than 4.2 μm) takes up a percentage of 50%. According to the Food and Drug Administration’s (FDA) guidance documents, the nebulized medication particles must be less than 5 μm in MMAD in order to provide enough therapeutic effects. The test results showed that the formed nebulizer plate developed in this paper possesses the ability to produce uniformly distributed ultrafine particles.

## 6. Conclusions

To fabricate desirable HAR ultrafine tapered holes for some applications, a nontraditional over-growth electroforming process was proposed. The evolution of the geometric shape of the holes being formed was investigated numerically and experimentally, and the correlations between the outlet size of the tapered hole and the key formation parameters were established. The application effect using the electroformed HAR ultrafine tapered holes as the medical nebulization nozzles was evaluated. Some conclusions were made as follows.

(1) The geometric shape and dimensions of the tapered holes are highly dependent on the formation parameters, including the film pattern thickness, film pattern diameter, and metallic deposit thickness, but are hardly affected by the spacing between two neighboring film patterns.
(2) The outlet diameter of the tapered hole becomes smaller with reducing the film pattern thickness when the metallic deposit thickness is kept constant, and these two parameters show a linear relation: *Y_outlet diameter_* = 2.93 + 0.6*X_pattern thickness_*.
(3) The outlet diameter of the tapered hole increases with the increasing film pattern diameter when the metallic deposit thickness is kept constant, and these two parameters also show a linear relation: *Y_outlet diameter_* =-5.95 + 0.315*X_pattern diameter_*.
(4) The over-growth electroforming process is highly effective and efficient in fabricating a perforated metallic plate with HAR ultrafine tapered holes.

## Figures and Tables

**Table 1:** The main parameters for the simulation operations.

Items (Unit)	Value
Volume electrochemical equivalent (μm·dm^2^·(A·h)^−^^1^)	12.4
Ni^2+^ initial concentration (mol·m^−^^3^)	500
Voltage (V)	3
Electrolyte conductivity (S·m^−^^1^)	5
Gap between the electrodes (mm)	200
Film pattern diameter (μm)	30, 50, 70
Film pattern thickness (μm)	1, 3, 5
Spacing between two-neighboring film patterns (μm)	75, 100, 150
Time (s)	30
Time step (s)	1
Volume electrochemical equivalent (μm·dm^2^·(A·h)^−1^)	12.4

**Table 2:** Compositions of electrolyte and electrodeposition parameters.

Compositions of Electrolyte and Electrodeposition Parameters (Unit)	Value
Ni(NH_2_SO_3_)_2_·4H_2_O (g·L^−^^1^)	360
C_12_H_25_SO_4_Na (g·L^−^^1^)	0.05
NiCl_2_·7H_2_O (g·L^−^^1^)	10
H_3_BO_3_ (g·L^−^^1^)	40
pH	4.5
Temperature (°C)	55
Voltage (V)	3
